# Effects of ACT Out! Social Issue Theater on Social-Emotional Competence and Bullying in Youth and Adolescents: Cluster Randomized Controlled Trial

**DOI:** 10.2196/25860

**Published:** 2021-01-06

**Authors:** Jon Agley, Mikyoung Jun, Lori Eldridge, Daniel L Agley, Yunyu Xiao, Steve Sussman, Lilian Golzarri-Arroyo, Stephanie L Dickinson, Wasantha Jayawardene, Ruth Gassman

**Affiliations:** 1 Prevention Insights, Department of Applied Health Science School of Public Health Indiana University Bloomington Bloomington, IN United States; 2 School of Social Work Indiana University Bloomington and Indiana University-Purdue University Indianapolis Indianapolis, IN United States; 3 Department of Preventive Medicine Keck School of Medicine University of Southern California Los Angeles, CA United States; 4 Department of Psychology Dana and David Dornsife College of Letters, Arts and Sciences University of Southern California Los Angeles, CA United States; 5 School of Social Work University of Southern California Los Angeles, CA United States; 6 Biostatistics Consulting Center School of Public Health Bloomington Indiana University Bloomington Bloomington, IN United States

**Keywords:** cyberbullying, bullying, social-emotional learning, SEL, social-emotional competence, RCT, randomized controlled trial, outcome, emotion, bully, prevention, school, intervention, assessment, effectiveness, implementation, fidelity, reception, children, young adults, adolescents

## Abstract

**Background:**

Schools increasingly prioritize social-emotional competence and bullying and cyberbullying prevention, so the development of novel, low-cost, and high-yield programs addressing these topics is important. Further, rigorous assessment of interventions prior to widespread dissemination is crucial.

**Objective:**

This study assesses the effectiveness and implementation fidelity of the ACT Out! Social Issue Theater program, a 1-hour psychodramatic intervention by professional actors; it also measures students’ receptiveness to the intervention.

**Methods:**

This study is a 2-arm cluster randomized control trial with 1:1 allocation that randomized either to the ACT Out! intervention or control (treatment as usual) at the classroom level (n=76 classrooms in 12 schools across 5 counties in Indiana, comprised of 1571 students at pretest in fourth, seventh, and tenth grades). The primary outcomes were self-reported social-emotional competence, bullying perpetration, and bullying victimization; the secondary outcomes were receptiveness to the intervention, implementation fidelity (independent observer observation), and prespecified subanalyses of social-emotional competence for seventh- and tenth-grade students. All outcomes were collected at baseline and 2-week posttest, with planned 3-months posttest data collection prevented due to the COVID-19 pandemic.

**Results:**

Intervention fidelity was uniformly excellent (>96% adherence), and students were highly receptive to the program. However, trial results did not support the hypothesis that the intervention would increase participants’ social-emotional competence. The intervention’s impact on bullying was complicated to interpret and included some evidence of small interaction effects (reduced cyberbullying victimization and increased physical bullying perpetration). Additionally, pooled within-group reductions were also observed and discussed but were not appropriate for causal attribution.

**Conclusions:**

This study found no superiority for a 1-hour ACT Out! intervention compared to treatment as usual for social-emotional competence or offline bullying, but some evidence of a small effect for cyberbullying. On the basis of these results and the within-group effects, as a next step, we encourage research into whether the ACT Out! intervention may engender a bystander effect not amenable to randomization by classroom. Therefore, we recommend a larger trial of the ACT Out! intervention that focuses specifically on cyberbullying, measures bystander behavior, is randomized by school, and is controlled for extant bullying prevention efforts at each school.

**Trial Registration:**

Clinicaltrials.gov NCT04097496; https://clinicaltrials.gov/ct2/show/NCT04097496

**International Registered Report Identifier (IRRID):**

RR2-10.2196/17900

## Introduction

### ACT Out! Social Issue Theater

The ACT Out! Ensemble was founded in 1995 and is currently operated by Claude McNeal Productions (CMP), a professional theater troupe incorporated as a not-for-profit [1]. The ensemble uses scripted content (scenarios) generated to meet an audience’s needs and transforms it into improvisational, interactive theater performances. The actors deliver performances focused on a variety of topics salient to youth and adolescents, including bullying, diversity, inclusion, and substance use [1]. A facilitator pauses the action at the end of each scenario and enables discussion between attendees and the actors, who remain in character for the discussion [1]. For example, a scenario about bullying may instruct the group to create a scene with “an example of a male student making a female student physically uncomfortable.” After the actors present an interpretation of that scene, the facilitator pauses the action and asks the audience “whether the male character meant to make the female character uncomfortable,” with the actors participating, in character, in the audience discussion.

The premise behind the ACT Out! Social Issue Theater intervention, outlined in the trial protocol, is that dramatic performances can enable emotional catharsis, thereby allowing new ways of feeling and thinking about behaviors and attitudes [2]. In other words, there is likely a difference between students discussing or attending a lecture on an issue like bullying in their own lives (where their own identity has weight and affects perceptions) and students’ emotional responses to a scenario that seems real and familiar (eg, bullying) but that is occurring with characters rather than with themselves or their peers. The latter case would theoretically enable students to process their reactions to bullying separately from their own or their peers’ identities. This is facilitated by the high caliber of talent involved in the intervention performances; shows by CMP have received positive reviews from, among other venues, the *New York Times*, *NBC*, and *Time* magazine [3]. For this trial, CMP developed and revised 15 vignettes (5 per participating grade level) addressing bullying and cyberbullying using principles of social-emotional learning (SEL) and reviewed the content with the research team. A presentation of the 5 psychodramatic vignettes was planned to last approximately 1 hour, including student interaction with the characters, and these performances constituted the intervention for this trial. This paper describes a rigorous evaluation, through a cluster randomized controlled trial, of the ACT Out! Ensemble’s theater performance, addressing bullying and SEL.

### Social and Emotional Learning

In the United States and internationally, schools, school-based professionals, and policymakers have begun focusing on positive development models as a means of addressing the numerous, complex, and detrimental behavioral patterns and associated outcomes (eg, bullying, mental health problems, self-harm, and substance use) observed among youth and adolescents [4]. Such approaches deliberately avoid a deficit approach (“fixing what is wrong”) and emphasize the development of assets or protective factors in youth. Among the most common and conceptually similar positive development models are positive youth development [5], which emphasizes skills development, healthy relationship development, supportive community systems, and SEL. SEL focuses on the instruction of skills such as social problem-solving, recognizing emotions in others, and emotional self-regulation [6]. There have been numerous SEL programs implemented and evaluated in schools in recent years; a summary of over 300 studies contained in 4 meta-analyses identified generally positive short-term outcomes [7] across multiple domains (eg, substance use). In general, performances by the ACT Out! Ensemble are structured to model aspects of SEL, such as healthy relationships, regardless of the additional topic being addressed (eg, bullying). We have summarized additional content related to SEL and social-emotional competence (SEC) as it pertains to this study in our published protocol [2].

### Bullying and Victimization

School bullying is frequently mentioned among the detrimental behaviors addressed by SEL programs [8,9]. Bullying is an unfortunate reality for youth attending US schools; a meta-analysis of 80 studies (youth aged 12 to 18 years) found a 35% student-level prevalence of traditional bullying and a 15% prevalence of cyberbullying [10]. Being bullied in childhood and adolescence has been associated with long-term, negative consequences that persist into midlife in areas such as mental and general physical health and lower socioeconomic status attainment [11]. Further, bullying victimization appears likely to cause notable increases in anxiety and depression among those victimized [12].

A recent meta-analysis of traditional bullying identified 65 school-based bullying prevention programs, but only 8 had been evaluated more than once [13]. In general, such programs tend to be slightly more effective in reducing bullying perpetration and less effective in reducing victimization [14]. Mean values from meta-analyses have been somewhat consistent in terms of victimization, reporting reductions of 15-16% [14] and 17-20% [15]. A separate meta-analysis, focused only on cyberbullying, reported a mean reduction in victimization of 14-15% [16]. However, a meta-analysis of victimization studies limited to randomized controlled trials with a high level of rigor reported a rather small effect size (standard mean difference of -.09) [17]. Each of the cited meta-analyses note high levels of heterogeneity in the types of programs and outcomes across included programs and studies; importantly, these differences extended to study design and rigor, with some scholars noting that beneficial effects appear to be weaker when measured as part of randomized controlled trials [18].

As one might expect, reductions in victimization tend to be larger for more intensive and multifaceted programs, but implementing such programs can be both expensive and complicated [11,19,20]. For example, a summary of nontargeted (general population), relatively efficacious bullying prevention programs for US elementary schools found that program durations ranged from 11 weeks to 3 years [21]. Even efficacious programs described as “brief” prevention curricula can last 1 week or more and involve multiple interlocking components [22]. Further, several programs that have reported favorable results in efficacy trials have not always produced the same results in effectiveness (“real world”) trials, potentially due to issues with implementation fidelity and existing, confounding antibullying programming [23,24]. In concluding their report from a recent effectiveness trial, Rapee et al [24] noted, “clearly, producing a sizeable impact on school-based victimization is extremely difficult.” Therefore, there is a demonstrated need for inexpensive, simple-to-implement bullying prevention programming, but achieving positive outcomes from such interventions is likely to be especially challenging. For this reason, we believe that innovative or out-of-the-box strategies to address bullying merit serious consideration.

### Psychodrama and Professional Acting as Innovation

Given the difficulty in addressing school-based bullying with lengthy and multipartite curricula, one might wonder why a short (1-hour) dramatic performance would be hypothesized to have even a short-term effect on SEC or bullying. A small body of literature has examined psychodrama as a prevention or behavior-change mechanism in youth, but these studies have covered diverse behaviors [25], have involved multiple, separate components such as teacher training [26], or have used students or school employees rather than professional actors as *dramatis personae* [27]. ACT Out! Social Issue Theater is different than each of these examples because it uses trained, professional actors and requires no involvement from schools outside of planning the visit (when implemented outside of a study). We were unable to find a precedent for this intervention structure in the literature.

Our decision to analyze this intervention was based on our a priori understanding of the value this brief intervention might yield as well as the remarkable community- and school-level support for the program. Prior to this study, more than 500,000 individuals had viewed a performance by the ACT Out! Ensemble [1], providing a notable depth of informal, qualitative evidence supporting the program. Uncontrolled evaluations of the program from 2015 also suggested substantive behavioral benefits [1]. Thus, given the importance of both SEL and bullying prevention in schools, and the unique position occupied by the ACT Out! Ensemble, we determined that an independently conducted, randomized controlled trial of this intervention was a valuable contribution to the prevention literature.

### Study Objectives

This study primarily aims to assess whether a 1-hour exposure to ACT Out! Social Issue Theater is superior to treatment as usual for developing SEC and reducing bullying (both bullying behavior and victimization) in elementary, middle, and high school students at a 2-week posttest. Secondarily, the study aims to determine whether the same intervention is superior to treatment as usual in developing specific subdomains of SEC (social awareness, emotion regulation, relationship skills, and responsible decision making) among middle and high school students at a 2-week posttest. Finally, the study also aims to assess student receptivity to ACT Out! Social Issue Theater using previously validated measures indicating student agreement with positive (eg, “enjoyable”) and negative (eg, “boring”) adjectives. Additional details are available in the trial protocol [2]. All outcomes were measured at the individual participant level, but randomization occurred at the cluster level (classroom) because performances are intended to be delivered to groups and because research literature [23,24] has indicated that pre-existing school-level programs addressing bullying and SEC often vary between schools and may contribute to statistical noise in randomized trials using school as the cluster (eg, treatment as usual may not be consistent between schools).

## Methods

### Trial Design

The ACT Out! trial was a proof-of-concept cluster randomized superiority trial with 2 groups and 1:1 allocation. The unit of measurement was individual students, but the unit of randomization was the classroom, stratified by school (with 1 exception, Multimedia Appendix 1). For each school, half of the classrooms were randomly assigned to the intervention arm and half to the control arm. Schools with an odd number of classrooms had a single classroom randomly selected for exclusion (though if the school requested, that classroom was permitted to complete the survey for appearance’s sake, and the results were then discarded by the study team).

### Participants and Recruitment

The ACT Out! trial was conducted among 12 public and charter schools in Indiana: 4 schools in Marion County, 3 in Ripley County, 2 in Boone County, 2 in Lawrence County, and 1 in Monroe County. For reasons described in the protocol, clusters were selected only from grades 4, 7, and 10 [2]. All students in the selected classrooms and schools were eligible to participate. As planned, we recruited schools until meeting a threshold of approximately 80 participating classrooms (around 1594 students) across both conditions.

Schools were selected based on their willingness to participate in the project as described, which included classroom-level randomization and inclusion of all eligible classrooms in the study’s allocation processes. Authorizing officials for schools or school corporations were required to provide a signed letter of agreement prior to participating in the study. At the individual level, the project used a waiver of parental consent (opt out), as approved by the institutional review board. Parents and legal guardians were permitted to review study procedures and were provided with a description of the study a minimum of 2 weeks prior to any individual-level interaction with subjects, along with instructions for how to opt out; students, their parents, and their guardians all had the ability to opt a student out from participating either formally or by survey noncompletion. The rationale for this approach was a combination of the low risk posed by the study as well as the desire to avoid unintentional exclusion of underrepresented minorities and high-risk populations, as described in the protocol [2]. This study and all consent procedures were carried out according to, and approved by, the Indiana University Institutional Review Board.

### Intervention

#### ACT Out! Social Issue Theater

The intervention was a psychodramatic, improvisational performance that was delivered to classrooms (separately, except in 1 case where 2 small classrooms attended together) by members of the CMP professional theater company. Interventions were scheduled to last approximately 1 hour and were delivered during the school day. Each 1-hour performance consisted of 5 vignettes focused on bullying and cyberbullying and was designed to be interactive; after each scenario, the student audience was invited to converse with the performers, who remained in character. In each case, a moderator from CMP also managed the overall performance (eg, calling on students to ask questions of the characters). While the scenarios were improvisational in nature, they were designed to remain true to core concepts that were prespecified and agreed upon by CMP and the research team (eg, the identity of the characters, the nature of the conflict, and methods of bullying). To ensure this, fidelity data were captured from all performances (described in the *Quality Control*). The written specifications for each vignette, by grade level, are provided in Multimedia Appendix 2.

#### Treatment as Usual (Control)

Classrooms randomized to treatment as usual were provided with the preparation materials for the survey (see *Data Collection*) and completed the survey tools in the classroom during the school day (both at baseline and 2-week posttest). Students were not otherwise informed about the ACT Out! intervention by study personnel. Within schools, we were not aware of any systematic differences between intervention and control classrooms aside from the ACT Out! intervention itself, though schools themselves likely had different SEL and bullying programs at the school level (our statistical models incorporated random effects at both the classroom and school level).

### Outcomes

All measured outcomes were prespecified in the clinical trial registration and the published protocol [2], along with the rationale for their selection, and have been validated. Unfortunately, certain outcomes were not possible to collect due to the COVID-19 pandemic, which led to mandated school closures in the state during part of the data collection period (Multimedia Appendix 1). However, we do not have reason to suspect that the data we collected prior to closures were substantively affected.

Thus, this study collected the following 2 primary outcomes: (1) social-emotional competence and (2) bullying behavior and bullying victimization. Overall social-emotional competence was measured at baseline and at 2-week posttest using the Delaware Social-Emotional Competency Scale (DSECS-S) [28]. This scale prompted students to “Please read each statement and mark the response that best shows how much it is like you,” with response options of 1=*Not like me at all*, 2=*Not much like me*, 3=*Somewhat like me*, and 4=*Very much like me*. An example statement from the scale is, “I can control how I behave.” This scale demonstrated good internal consistency [intention-to-treat (ITT) pretest α=.78] for the study sample. Bullying behavior and experiences of being bullied (victimization) were measured at baseline and at 2-week posttest using the Bullying and Cyberbullying Scale for Adolescents (BCS-A) [29]. These questions measured the number of times (between 0 and 4+) in the past 2 weeks that students “bullied another school student” or, separately, “had been bullied.” These sections were further separated into subsections for “online/on the internet or mobile phones” (eg, cyberbullying behaviors) and “offline/face-to-face” (including physical, verbal, and relational behaviors).

Further, the study collected the following 2 secondary outcomes: (1) receptiveness to the intervention and (2) prespecified subanalyses of social-emotional competence for seventh- and tenth-grade students. Student receptivity to the intervention \was measured at 2-week posttest (intervention arm only) using questions to assess the degree to which they found the intervention to be enjoyable, interesting, a waste of time, boring, understandable, difficult to understand, believable, important, and helpful [30]. Social-emotional competence subdomains (social awareness, emotion regulation, relationship skills, and responsible decision-making) were measured at baseline and 2-week posttest (seventh and tenth grades only) using scales from the Washoe County School District Social-Emotional Competency Assessment (WCSD-SECA) [31]. These scales prompted students to “Please tell us how easy or difficult each of the following are for you,” with response options of 1=*Very difficult*, 2=*Difficult*, 3=*Easy*, and 4=*Very easy*. An example item from one scale is, “Getting along with my teachers.” Though the scales were previously developed to be reliable and valid [31], the items are relatively heterogeneous, perhaps contributing to their rather mediocre internal consistency with this sample [ITT pretest α=.57 (social awareness), .65 (emotion regulation), .68 (relationship skills), and .66 (responsible decision-making)].

### Sample Size

The rationale for choices made in preparing the sample size calculation is provided in the protocol [2]. We estimated the sample size required to detect a moderate effect (Cohen *d*=0.30) with a 2-sided significance of .05 and a power of .80 to be 340 participants. We estimated an intraclass correlation of 0.153 based on a prior school-based cluster study on cigarette smoking with a similar methodology [2] and assumed approximately 20 students per classroom, yielding a design effect of 3.907. We took the resultant estimate of 1328 students and multiplied it by 1.2 to account for attrition and potential loss of matched pairs due to survey matching procedures, producing the final sample size target of 1594 students across approximately 80 classrooms.

### Randomization and Allocation

#### Sequence Generation and Type of Randomization

Simple randomization occurred at the cluster level using a smartphone app produced by Random.org [32]. Randomization of clusters occurred within schools with a 1:1 allocation. In the specific instance where clusters first had to be created (a single school), the website version of Random.org was used to randomly assign students to evenly sized clusters and then to assign clusters to a study arm.

#### Concealment, Implementation, and Blinding

Since the generation of the allocation sequence was computerized, it was concealed to all members of the research team until the moment of assignment. Because of needs driven by school planning, there was some variability in the generation and assignment process. Decisions were made as follows: Schools that agreed to participate were asked to identify all clusters within the selected grade level (eg, fourth, seventh, or tenth). If schools were willing and able to accommodate it, allocation sequences were generated by the fidelity checker immediately prior to the intervention (eg, on-site, in the schools). However, most schools (10/12) were unable to accommodate this method. Subsequently, most schools were asked to list classrooms by a fixed characteristic (eg, the time the homeroom met, teacher’s name) at the time of school enrollment. One of the researchers generated a random sequence, applied it directly to classrooms, and shared the sequence with school administrators, identifying which classrooms would be allocated to which arm; the researcher asked the administrators not to share this information with teachers until necessary for planning efforts.

Consent was obtained from an administrative authority at participating schools at the time of enrollment and prior to randomization. Students and their parents or legal guardians were notified at least 2 weeks in advance of the intervention and provided an opportunity to opt out of the study but were not informed about their classroom’s allocation. Due to the nature of this study, blinding of participants, school officials, and researchers was not feasible. However, multiple independent statisticians were involved in conducting and reviewing analyses, and some were blinded to the meaning of study arm coding.

### Data Collection

#### Survey Administration

Once a school enrolled in the study, each classroom was provided with a study packet containing surveys and response forms, a manila envelope, a white envelope, and an administrator checklist [2]. Each classroom was also assigned a unique code consisting of the grade level, study arm, and a randomly generated cluster ID. This code was prefilled on the back of each survey form and on the front of each envelope to facilitate data quality control.

Classroom teachers administered the surveys by following the step-by-step administrator checklist. Surveys that were handed out to students were placed back in the manila envelope, regardless of whether they were completed, while extra surveys were placed in the white envelope (unused). The pretest was completed 0-3 days prior to the intervention, depending on school schedules and availability. The posttest was completed 14-27 days after the intervention, with most classrooms completing the posttest within 14 to 17 days, depending on school schedules and the ability to facilitate the posttest.

#### Quality Control

Data were collected using a customized form created with Scantron DesignExpert (Scantron) and scanned directly into a database with an Insight 700c scanner (Scantron) to avoid data entry errors. However, one of the survey matching elements required a handwritten response; these were typed manually into the database. To verify intervention fidelity, at least one individual who was not a member of the ACT Out! Ensemble attended every performance and documented the concordance between a prespecified checklist of elements for the intervention and the performance itself. These checklists were developed separately for each grade (since the scenarios vary) and are available in Multimedia Appendices 3-5. To establish coding reliability, a second individual attended performances for 6 clusters to conduct fidelity checks, and interrater reliability was computed (Multimedia Appendix 1).

### Survey Matching Procedures

This study required an anonymous procedure to match students’ surveys between pretest and posttest while remaining compliant with the requirements of the institutional review board. As described in the protocol [2], even recent meta-analyses had not identified a best-practice solution to such a dilemma [33]. Thus, the project team developed and used a novel anonymous matching procedure based on unique self-generated identification code elements and machine-assisted weighted matching. This approach was sufficiently complex that it required a separate full-length manuscript to articulate [34], and a complete description would extend well beyond the scope of this paper.

### Analytic Methods

#### Missing Data

Multiple imputation (MI) using the Markov Chain Monte Carlo approach was completed using SAS (version 9.4; SAS Institute), utilizing PROC MI and MIANALYZE with the assumption that data were missing at random. All variables that were collected were imputed for all analyses. Numbers of iterations were based on missingness in the per-protocol (PP) analysis, which had 1184 pretests and posttests, and thus 2368 surveys. Percent missingness ranged from a low of 1.22% to a high of 8.57%. SEC items not asked of fourth-grade students were present on 2078 surveys (removing 145 fourth-grade participants). Percent missingness within those variables ranged from 2.84% to 4.96%. Given this information, we selected 10 imputations for our analyses (integer greater than the missingness in the variable with the highest level of missingness, 10>8.57 [35]). Bias was also mitigated by presenting outcomes from 4 approaches.

#### Statistical Analyses

**Figure 1:**
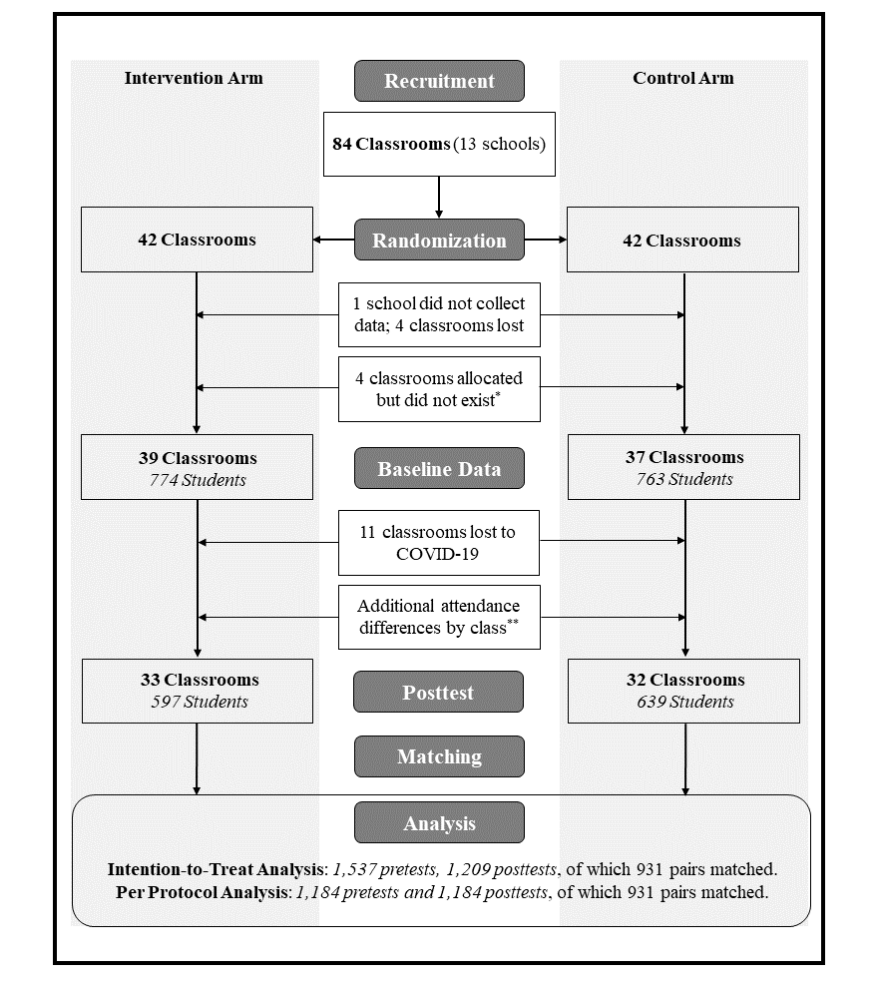
Consort flow diagram. *Classroom count was provided by schools and used to randomize clusters. When arriving to deliver interventions, it was discovered that 4 classrooms that had been allocated did not exist (n=3 schools). **Attendance differences by cluster are provided in supplemental material for the matching procedure paper [34].

All outcomes were continuous, so linear mixed models using restricted maximum likelihood were fitted for each analysis (SAS PROC MIXED) with repeated measures for each participant. The time of survey administration (pretest or posttest), study arm, and time and study arm interaction were treated as fixed effects. The interaction of time by study arm was the hypothesis test for causal effects (eg, intervention group improved significantly more than the control group). All analyses allowed for clustering of students within schools and classrooms as random intercepts to alleviate the issue of inflated standard errors and used Kenward-Rogers degrees of freedom approximation to account for the cluster randomized trial design. *P* values were 2-sided and treated as significant at .05 or less; however, in keeping with recommendations from the American Statistical Society, we did not use *P* values as the sole determinant of outcome importance. Instead, we provided the full dataset and analytic code, and interpreted the output based on a combination of effect size, clinical significance, standard errors, and significance [36]. Similarly, we produced 4 sets of output, the ITT analysis in which all data were analyzed in the arm to which they were randomized (with and without MI; 1537 pretest and 1209 posttest), and the PP analysis in which only data resulting from a completed protocol were analyzed (with and without MI; 1184 pretest and posttest), in accordance with reporting recommendations [37] (Figure 1). Notably, the 931 cases that were matched do not represent the totality of students who complied with the protocol but rather are those who provided internally consistent information for the variables used to match anonymous surveys. The PP analyses included an additional 253 individuals (1184 in total) who were likely to have completed both surveys, even though their specific surveys could not be reliably matched between time points. The 931 individuals were included as repeated measures in the analysis, and the remaining 253 were included with surveys that were unmatched between time points.

ITT has been suggested to more closely represent effectiveness while PP represents efficacy [38]. Thus, superiority randomized controlled trials typically emphasize ITT analyses with imputation. In this case, however, the preponderance of excluded data resulted predominantly from an unexpected global event (COVID-19) rather than intervention nonadherence. Being able to attribute attrition directly to an external factor unrelated to the study is rare in controlled studies. As a result, it is less clear to us that ITT better reflects the true findings than PP in this specific instance. Thus, we interpreted ITT and PP analyses in tandem, providing all data from each analysis. We felt that this approach, while more complex in terms of preparing written text, facilitated transparency in explicating the findings.

## Results

### Recruitment

The formal study start date was October 16, 2019, and interventions were delivered from November 6, 2019, to February 28, 2020. School recruitment was terminated in February once the anticipated numbers of clusters and participants reached the planned total. Initially, a total of 13 schools from across Indiana participated in the trial, comprising 84 classrooms for the eligible grade levels. Of those 84 classrooms, 42 were randomized to the intervention arm and 42 were randomized to the control arm. One school did not follow protocol and failed to correctly administer pretest surveys to either arm prior to intervention delivery, so it (4 classrooms) was summarily removed from the trial. In addition, 3 schools provided incorrect counts of classrooms to be randomized (3 control classrooms and 1 intervention classroom), so sequences were generated that included classrooms that did not exist. Upon discovery of this discrepancy, sequences and assignments were not altered because it would have affected allocation concealment. Thus, despite 1:1 allocation, the number of baseline classrooms was 76 (37 control and 39 intervention classrooms). Finally, an additional 11 classrooms (5 control and 6 intervention classrooms) at a single school were slightly delayed in completing posttests, and then schools were shut down for the academic year due to COVID-19 prior to data collection. Thus, the number of classrooms that completed posttests was 65 (32 control and 33 intervention). Table 1 shows the baseline characteristics of the 2 trial arms for all students who provided data, excluding blank surveys.

**Table 1:** Baseline sample characteristics of the study participants (n=1537).

Characteristic		Control group (n=763)		Intervention group (n=774)	
**Gender, n (%)**					
	Male		386 (51.8)		404 (52.9)
	Female		359 (48.2)		360 (47.1)
	Missing		18 (—^a^)		10 (—)
**Grade, n (%)**					
	Fourth		81 (10.6)		73 (9.4)
	Seventh		307 (40.2)		293 (37.9)
	Tenth		375 (49.2)		408 (52.7)
**Race, n (%)**					
	White		526 (72.2)		548 (73.1)
	African-American or Black		67 (9.2)		77 (10.3)
	Asian		14 (1.9)		11 (1.5)
	Native American or Alaskan Native		10 (1.4)		7 (0.9)
	Hawaiian or Pacific Islander		0 (0.0)		2 (0.3)
	Multiracial		74 (10.2)		61 (8.1)
	Other		38 (5.2)		44 (5.9)
	Missing		34 (—)		24 (—)
**Hispanic/Latino, n (%)**					
	Yes		92 (12.7)		87 (11.8)
	No		634 (87.3)		653 (88.2)
	Missing		37 (—)		34 (—)
**Bullying victimization^b^, mean (SD)**					
	Traditional (physical)		0.57 (0.88)		0.57 (0.93)
	Traditional (verbal)		1.13 (1.49)		1.20 (1.55)
	Traditional (relational)		0.78 (1.20)		0.82 (1.23)
	Cyber		0.48 (0.83)		0.56 (0.93)
**Bullying perpetration^b^, mean (SD)**					
	Traditional (physical)		0.25 (0.59)		0.24 (0.60)
	Traditional (verbal)		0.56 (1.05)		0.61 (1.13)
	Traditional (relational)		0.26 (0.74)		0.25 (0.71)
	Cyber		0.25 (0.61)		0.24 (0.59)
Social-emotional competence^c^, mean (SD)		3.20 (0.45)		3.18 (0.48)	
Self-awareness^d^, mean (SD)		2.94 (0.48)		2.90 (0.50)	
Emotion regulation^d^, mean (SD)		2.37 (0.61)		2.44 (0.62)	
Relationship skills^d^, mean (SD)		2.79 (0.52)		2.76 (0.55)	
Responsible decision-making^d^, mean (SD)		2.92 (0.54)		2.93 (0.55)	

^a^—: not available.

^b^Count variable scored from 0 (no instances of any exemplars of bullying in the category) to 4 (4 or more instances of every exemplar of bullying in the category) [29].

^c^Scored from 1 to 4, where 4 is the optimal score [31].

^d^Seventh- and tenth-grade students only; scored from 0 to 4, where 4 is the optimal score [28].

### Fidelity

Interrater reliability for fidelity coding was excellent (96.5% concordance). Fidelity to the intervention was high for all grades, though some seventh- and tenth-grade classrooms were not able to proceed through all 5 scenarios due to timing constraints. These instances were noted distinctly from fidelity because they resulted from longer-than-expected time completing the pretest survey immediately prior to the intervention, a factor that would not exist outside of the study. Fidelity was computed as the sum of all completed checkpoints for all clusters divided by the sum of all possible checkpoints for all clusters, within each grade. For the 5 fourth-grade classrooms, intervention fidelity was 100%. For the 15 seventh-grade classrooms, fidelity was 96.9%, but the fifth scenario was excluded for all clusters due to time constraints. For the 14 tenth-grade classrooms, fidelity was 98.4%, but 3 checkpoints for 1 classroom were excluded (fidelity checker was meeting with administrators), the third scenario was excluded for 2 clusters, the fourth scenario was excluded for 3 clusters, the fifth scenario was excluded for 3 clusters, and both the fourth and fifth scenarios were excluded for 1 cluster, all due to time constraints.

### Outcomes

#### Sociodemographic Characteristics

All baseline data for the study are provided in Table 1, sorted by study arm. No significant sociodemographic differences were observed between the control and intervention arms based on chi-square tests with unadjusted alpha (.05) and pairwise exclusion of cases with missing sociodemographic values. The overall study sample was mostly male (790/1509, 52.4%), non-Hispanic/Latino (1287/1466, 87.8%), and White (1074/1479, 72.6%). More participants were in seventh (600/1537, 39.0%) and tenth (782/1537, 50.9%) grades than in fourth grade (154/1537, 10.0%).

#### Primary Objective 1

In our protocol [2], we hypothesized that ACT Out! Social Issue Theater was superior to treatment as usual for the development of overall SEC in students enrolled in elementary, middle, and high schools, measured approximately 2 weeks postintervention using the DSECS-S. The data did not support this hypothesis; no clinically or statistically significant interactions were observed (although this was not a “clinical” study, we use the term “clinical significance” to indicate findings where the magnitude, in our opinion, might reasonably be inferred to be of interest or value to potential stakeholders).

#### Primary Objective 2

In our protocol [2], we hypothesized that ACT Out! Social Issue Theater was superior to treatment as usual for reducing frequency (count) of perpetration of and victimization from traditional bullying (physical, verbal, and relational) and cyberbullying, measured approximately 2 weeks postintervention. These findings were complex. To interpret clinical significance, it is important to know that bullying scores are mean values based on count data of multiple bullying behaviors within a category, and so interpreting outcomes is different than for an attitudinal scale. For example, an individual who scored 1 for physical bullying victimization would need to have reported 1 instance of *each* of the 4 types of physical bullying victimization that compose that scale [2,29]. Similar interpretation applies to verbal (2 types), relational (2 types), and cyber (5 types) bullying. For example, a baseline bullying score of 0.568 (physical victimization) means that the average student reported experiencing more than 2 instances of physical bullying behaviors in the past 2 weeks (computed as .568*4). This should be taken into account when interpreting bullying outcomes.

There was limited causal, clinically significant evidence of small reductions (assessed via interaction effects of time by study arm) for cyberbullying victimization (favoring the intervention arm) and physical bullying victimization (favoring the control arm). For cyberbullying, reductions in the intervention arm victimization score ranged from -0.08 (*P*=.011, ITT with MI) to -0.13 (*P*<.001, PP without MI). This corresponded to mean cyberbullying victimization reductions of 0.40 to 0.65 instances/2 weeks, where the interaction term comparing the reduction in the intervention arm to the control arm was marginally significant in the PP analysis without MI (*P*=.067) but was increasingly nonsignificant in other models, ranging up to *P*=.301 for ITT with MI. For physical bullying, reductions in the control arm victimization score ranged from -0.13 (*P*<.001, ITT with MI; interaction *P*=.013) to -0.14 (*P*<.001, PP without MI; interaction *P*=.062). This corresponded to mean physical bullying victimization reductions of 0.52 to 0.56 instances/2 weeks. There was also limited evidence of a small effect of similar magnitude for increased physical bullying perpetration via the interaction effects. Increases in the intervention arm perpetration score ranged from 0.06 (*P*=.013, interaction *P*=0.060; ITT with MI) to 0.08 (*P*=.005, interaction *P*=0.032; ITT without MI), corresponding to increased perpetration of 0.24 to 0.32 instances/2 weeks, while the control arm did not have significant increases. However, only the ITT without MI model showed significance.

Finally, we observed an overall decrease in bullying victimization across pooled study participants (both arms). Findings were fairly uniform across models, so we provide only the most conservative (ITT with MI) outcomes in this paper. This included small-to-moderate, clinically significant overall reductions in physical (as above), verbal (control: -0.29, *P*<.001; intervention: -0.29, *P*<.001), and relational (control: -0.15, *P*=.001; intervention: -0.15, *P*=.003) bullying victimization. These corresponded to reductions of 0.58 (verbal) and 0.30 (relational) mean victimization instances/2 weeks. There was also some evidence of a small or moderate overall decrease in verbal bullying perpetration (control: -0.10, *P*=.023; intervention: -0.12, *P*=.005), corresponding to a reduction of 0.20-0.24 mean verbal bullying perpetration instances/2 weeks. These findings were not causal (ie, they were not observed differentially for the intervention clusters) and do not directly support the original hypothesis for this objective (except, potentially, for cyberbullying victimization); however, we believe that they do provide some favorable evidence for the program. Table 2 shows the ITT study outcomes, and Table 3 shows the PP study outcomes.

**Table 2:** Intention-to-treat study outcomes with standard errors.

Variable (# observations)			Control			Intervention				Interaction		
			Pretest, mean (SE), n=763	Posttest, mean (SE), n=614	*P* value of difference		Pretest, mean (SE), n=774	Posttest, mean (SE), n=595	*P* value of difference		Difference in differences (SE)	*P* value
**Linear Mixed Models (no imputation)**												
	**Bullying victimization**											
		Physical (2623)	0.60 (0.05)	0.46 (0.05)	<.001		0.58 (0.05)	0.56 (0.05)	.514		0.11 (.05)	.031
		Verbal (2585)	1.16 (0.08)	0.85 (0.08)	<.001		1.24 (0.08)	0.94 (0.08)	<.001		0.02 (.08)	.852
		Relational (2569)	0.82 (0.06)	0.65 (0.07)	<.001		0.83 (0.06)	0.68 (0.07)	.003		0.02 (.07)	.741
		Cyber (2622)	0.49 (0.05)	0.45 (0.05)	.210		0.58 (0.05)	0.47 (0.05)	.001		-0.07 (.05)	.141
	**Bullying perpetration**											
		Physical (2604)	0.28 (0.04)	0.28 (0.04)	.860		0.26 (0.04)	0.34 (0.04)	.005		0.08 (.04)	.032
		Verbal (2576)	0.59 (0.05)	0.50 (0.06)	.022		0.62 (0.05)	0.50 (0.06)	.004		-0.03 (.06)	.636
		Relational (2570)	0.28 (0.04)	0.30 (0.04)	.516		0.28 (0.04)	0.30 (0.04)	.387		0.01 (.04)	.860
		Cyber (2595)	0.27 (0.03)	0.28 (0.03)	.623		0.26 (0.03)	0.30 (0.03)	.108		0.03 (.04)	.412
	**Social-emotional competence (2699)**		3.19 (0.02)	3.17 (0.03)	.182		3.17 (0.02)	3.15 (0.03)	.334		0.01 (.03)	.810
		Social awareness (2384)	2.94 (0.02)	2.93 (0.03)	.847		2.91 (0.02)	2.92 (0.03)	.464		0.02 (.03)	.510
		Emotion regulation (2374)	2.38 (0.03)	2.51 (0.03)	<.001		2.43 (0.03)	2.54 (0.03)	<.001		-0.02 (.04)	.559
		Relationship skills (2366)	2.78 (0.03)	2.80 (0.03)	.315		2.75 (0.03)	2.79 (0.03)	.193		0.01 (.03)	.813
		Responsible decision- making (2355)	2.91 (0.03)	2.95 (0.03)	.148		2.92 (0.03)	2.98 (0.03)	.006		0.02 (.03)	.460
**Multiple imputation analyses (2746)**												
	**Bullying victimization**											
		Physical	0.61 (.05)	0.48 (.05)	<.001		0.61 (.05)	0.60 (.05)	.830		0.13 (.05)	.013
		Verbal	1.18 (.08)	0.89 (.08)	<.001		1.28 (.07)	0.99 (.08)	<.001		0.00 (.09)	.966
		Relational	0.84 (.06)	0.69 (.06)	.001		0.89 (.06)	0.74 (.07)	.003		0.01 (.07)	.907
		Cyber	0.51 (.05)	0.47 (.05)	.270		0.60 (.05)	0.52 (.05)	.011		-0.05 (.05)	.309
	**Bullying perpetration**											
		Physical	0.30 (.03)	0.29 (.04)	.928		0.30 (.03)	0.36 (.04)	.013		0.07 (.04)	.060
		Verbal	0.61 (.05)	0.51 (.06)	.023		0.67 (.05)	0.55 (.06)	.005		-0.03 (.06)	.661
		Relational	0.30 (.04)	0.33 (.04)	.353		0.31 (.04)	0.34 (.04)	.239		0.01 (.04)	.844
		Cyber	0.28 (.03)	0.29 (.03)	.726		0.29 (.03)	0.32 (.03)	.148		0.03 (.04)	.430
	**Social-emotional competence**		3.19 (.02)	3.17 (.03)	.209		3.17 (.02)	3.15 (.03)	.386		0.01 (.03)	.814
		Social awareness	2.94 (.02)	2.93 (.02)	.732		2.91 (.02)	2.92 (.03)	.446		0.02 (.03)	.426
		Emotion regulation	2.38 (.03)	2.52 (.03)	<.001		2.43 (.03)	2.55 (.03)	<.001		-0.02 (.04)	.672
		Relationship skills	2.78 (.03)	2.80 (.03)	.295		2.75 (.03)	2.78 (.03)	.182		0.01 (.03)	.815
		Responsible decision- making	2.91 (.03)	2.95 (.03)	.142		2.91 (.03)	2.98 (.03)	.005		0.03 (.03)	.327

**Table 3:** Per-protocol study outcomes with standard errors.

Variable (# observations)				Control			Intervention			Interaction		
			Pretest, mean (SE), n=603		Posttest, mean (SE), n=603	*P* value of difference	Pretest, mean (SE), n=581	Posttest, mean (SE), n=581	*P* value of difference		Difference in differences (SE)	*P* value
**Linear Mixed Models (no imputation)**												
	**Bullying victimization**											
		Physical (2285)	0.61 (.05)		0.47 (.05)	<.001	0.63 (.05)	0.59 (.06)	.312		0.10 (.05)	.062
		Verbal (2252)	1.19 (.08)		0.87 (.08)	<.001	1.30 (.08)	0.98 (.08)	<.001		0.00 (.09)	.991
		Relational (2239)	0.82 (.07)		0.65 (.07)	<.001	0.89 (.07)	0.72 (.07)	.001		0.00 (.07)	.953
		Cyber (2285)	0.47 (.05)		0.44 (.05)	.292	0.63 (.05)	0.50 (.05)	<.001		-0.09 (.05)	.067
	**Bullying perpetration**											
		Physical (2272)	0.27 (.04)		0.27 (.04)	.954	0.27 (.04)	0.34 (.03)	.014		0.06 (.04)	.082
		Verbal (2245)	0.57 (.06)		0.49 (.06)	.060	0.67 (.06)	0.52 (.06)	.001		-0.06 (.06)	.302
		Relational (2244)	0.26 (.04)		0.29 (.04)	.314	0.30 (.04)	0.31 (.04)	.633		-0.02 (.04)	.725
		Cyber (2263)	0.25 (.04)		0.27 (.04)	.388	0.27 (.04)	0.31(.04)	.210		0.01 (.04)	.757
	**Social-emotional competence (2336)**		3.18 (.03)		3.15 (.03)	.198	3.16 (.03)	3.14 (.03)	.360		0.01 (.03)	.808
		Social awareness (2043)	2.94 (.03)		2.93 (.03)	.640	2.92 (.03)	2.93 (.03)	.635		0.02 (.03)	.510
		Emotion regulation (2034)	2.40 (.03)		2.52 (.03)	<.001	2.42 (.03)	2.54 (.03)	<.001		-0.00 (.04)	.944
		Relationship skills (2029)	2.77 (.03)		2.79 (.03)	.348	2.73 (.03)	2.77 (.03)	.065		0.02 (.03)	.588
		Responsible decision- making (2024)	2.91 (.04)		2.94 (.04)	.175	2.92 (.04)	2.98 (.04)	.007		0.03 (.03)	.400
**Multiple imputation analyses (2368)**												
	**Bullying victimization**											
		Physical	0.61 (.05)		0.48 (.05)	<.001	0.64 (.06)	0.62 (.05)	.568		0.11 (.05)	.036
		Verbal	1.20 (.08)		0.91 (.08)	<.001	1.33 (.08)	1.01 (.08)	<.001		-0.02 (.09)	.817
		Relational	0.83 (.07)		0.68 (.07)	.002	0.93 (.07)	0.76 (.07)	.001		-0.01 (.07)	.840
		Cyber	0.48 (.05)		0.46 (.05)	.453	0.63 (.05)	0.54 (.05)	.007		-0.07 (.05)	.154
	**Bullying perpetration**											
		Physical	0.27 (.04)		0.28 (.04)	.856	0.29 (.04)	0.35 (.04)	.023		0.05 (.04)	.125
		Verbal	0.57 (.06)		0.49 (.06)	.072	0.70 (.06)	0.55 (.06)	.002		-0.06 (.06)	.332
		Relational	0.27 (.04)		0.31 (.04)	.180	0.32 (.04)	0.34 (.04)	.374		-0.01 (.04)	.767
		Cyber	0.26 (.03)		0.28 (.03)	.400	0.29 (.04)	0.32 (.04)	.251		0.01 (.04)	.813
	**Social-emotional competence**		3.18 (.03)		3.15 (.03)	.219	3.16 (.03)	3.15 (.03)	.500		0.01 (.03)	.732
		Social awareness	2.94 (.03)		2.93 (.03)	.609	2.92 (.03)	2.93 (.03)	.591		0.02 (.03)	.459
		Emotion regulation	2.40 (.03)		2.53 (.03)	<.001	2.41 (.03)	2.55 (.03)	<.001		0.01 (.04)	.881
		Relationship skills	2.77 (.03)		2.79 (.03)	.354	2.72 (.03)	2.76 (.03)	.118		0.02 (.03)	.637
		Responsible decision-making	2.91 (.03)		2.94 (.03)	.171	2.91 (.03)	2.98 (.03)	.006		0.04 (.03)	.296

#### Secondary Objective 1

Table 4 shows student perception of the ACT Out! intervention. Students were highly receptive to the ACT Out! performance. Merging affirmative responses (both “Yes” and “YES!,” or, for negative questions, “No” and “NO!”), students found that the intervention was enjoyable (443/537, 82.5%), interesting (429/512, 83.8%), understandable (433/521, 83.1%), believable (433/517, 83.8%), important (417/506, 82.4%), and helpful (401/513, 78.2%). They also found that it was *not* a waste of time (424/518, 81.9%), boring (406/511, 79.5%), or difficult to understand (444/511, 86.9%).

**Table 4:** Perceptions of ACT Out! intervention. (Percentages may not add exactly to 100 due to rounding.)

Variable (# observations)		Values, n (%)
**Enjoyable (537)**		
	NO!	46 (8.6)
	No	48 (8.9)
	Yes	235 (43.8)
	YES!	208 (38.7)
**Interesting (512)**		
	NO!	34 (6.6)
	No	49 (9.6)
	Yes	236 (46.1)
	YES!	193 (37.7)
**Waste of time (518)**		
	NO!	230 (44.4)
	No	194 (37.5)
	Yes	56 (10.8)
	YES!	38 (7.3)
**Boring (511)**		
	NO!	227 (44.4)
	No	179 (35.0)
	Yes	64 (12.5)
	YES!	41 (8.0)
**Understandable (521)**		
	NO!	36 (6.9)
	No	52 (10.0)
	Yes	241 (46.3)
	YES!	192 (36.9)
**Difficult to understand (511)**		
	NO!	245 (48.0)
	No	199 (38.9)
	Yes	43 (8.4)
	YES!	24 (4.7)
**Believable (517)**		
	NO!	45 (8.7)
	No	39 (7.5)
	Yes	234 (45.3)
	YES!	199 (38.5)
**Important (506)**		
	NO!	36 (7.1)
	No	53 (10.5)
	Yes	202 (39.9)
	YES!	215 (42.5)
**Helpful (513)**		
	NO!	47 (9.2)
	No	65 (12.7)
	Yes	204 (39.8)
	YES!	197 (38.4)

#### Secondary Objective 2

As planned, we conducted secondary analyses to see whether the intervention was superior to treatment as usual for specific subdomains of SEC measured for seventh- and tenth-grade students only (social awareness, emotion regulation, relationship skills, and responsible decision-making) using the WCSD-SECA. No notable or significant interaction effects were observed. However, all pooled students (across conditions) reported a small, statistically significant increase in emotion regulation. In the ITT model with MI, this was 0.14 (*P*<.001) for the control arm and 0.12 (*P*<.001) for the intervention arm. As above, such effects do not support causal attribution to the intervention.

## Discussion

### Brief Summary

The ACT Out! Social Issue Theater trial was a prespecified and preregistered cluster randomized controlled trial conducted by a research team external to the program developers and supported by additional analysts who both were independent of the research team and the developers. Study findings were mixed.

### Implementation Fidelity

For school-based programs, implementation fidelity tends to be inconsistently documented and highly variable [39]. There is some evidence that achieving good fidelity may require intensive training of those delivering the intervention (eg, teachers, [40]) and may depend on school- and teacher-level variables [41]. Since the ACT Out! intervention was an improvisational and interactive intervention by actors, there was little precedent as to whether implementation fidelity would be achievable. We found that when provided with guidelines for core content elements, the professional actors in CMP were able to deliver nearly all (96.9% to 100%) prespecified content (Multimedia Appendices 3-5), even accounting for variance in student responses and different actors playing different roles, distinguishing this intervention from many school-based interventions. However, ACT Out! performances were short in duration and the actors had the delivery of the performance as their primary purpose; in contrast, teachers and schools must balance many different requirements simultaneously, involving the delivery of multiple sessions, and so high fidelity is conceptually reasonable to expect and may be a benefit of programs of this type.

### Student Receptivity

The degree to which students report enjoying a program or finding it to be realistic and engaging can be interpreted as an indicator of program quality and is a common component of process evaluation [30]. The ACT Out! intervention was very positively received by students across all specified metrics. Although not an indicator of emotional *competence*, we infer that this may be interpreted as a partial measure of emotional *response* to the intervention.

### Assessment of Effects on SEC

One of our primary hypotheses was that the ACT Out! intervention would improve students’ SEC in the short-term; this was suspected to be the mechanism through which the proposed emotional catharsis of psychodrama [2] could be measured. This study did not support that hypothesis, with analyses demonstrating neither statistical nor clinical significance, except improvement in emotion regulation regardless of treatment condition for seventh- and tenth-grade students.

During the project kickoff meeting, individuals at CMP expressed concern about quantitatively measuring SEC, at one point asking the research team, “How do you measure the sunrise?” We collected SEC data using 2 different tools that approached SEC in complementary ways [28,31]; however, optimal measurement of SEC remains a topic of debate, even among the national SEL workgroup [42]. Thus, we note (as with all such measurement) that the trial did not definitively find that the ACT Out! intervention had not engendered SEC development; rather, it found that SEC, as measured by the DSECS-S and WCSD-SECA (2 validated tools), was not affected by the intervention. It does not necessarily follow that those tools are the optimal or correct ways to measure students’ responses to the ACT Out! intervention (indeed, WCSD-SECA subscore reliability for this sample was suboptimal). It may also be the case that there was a partial ceiling effect [43] on overall SEC, as the baseline scores were relatively high: 3.202 and 3.176 for control and intervention groups, respectively, on a scale from 1-4, potentially making improvement from any source more difficult to achieve and measure. Given that all schools in the state already are required to offer SEL programming, this may also suggest that an intervention such as this would more appropriately be tested with subgroups of individuals who have lower baseline SEC (eg, not as a universal SEL program, but as an indicated program).

### Assessment of Effects on Bullying

We hypothesized that the classrooms viewing the ACT Out! intervention would report reduced bullying victimization and perpetration [both traditional (physical, verbal, relational) bullying and cyberbullying] relative to the control classrooms. There was little evidence for an effect on perpetration, though 1 of the 4 models indicated the potential for slight increases for physical bullying in the intervention arm. There was also some evidence of a small reduction in cyberbullying victimization attributable to the intervention, though not for the traditional forms of bullying. That cyberbullying might be influenced separately from traditional bullying is reasonable, as cyberbullying victimization is unique in several ways, such as where it occurs (in a digital space “outside” of school) and its ubiquity [44]. Further, some of the dramatic scenarios (2 of 5 for both seventh and tenth grades) explicitly focused on cyberbullying as opposed to traditional bullying.

There was also evidence that physical bullying victimization was lower among the control group than the intervention group at posttest, though this was not an iatrogenic effect since neither group reported increased victimization scores. This finding may have been related to the issue we discuss subsequently.

### Interpretation of Pooled Victimization Effects

Students reported clinically and statistically significant reductions in physical, verbal, and relational bullying victimization at posttest relative to pretest in aggregate (pooled across both study arms). This does not directly address the study hypotheses, as improvements were seen in students who did not participate in the intervention. However, this study examined students across 3 grade levels (fourth, seventh, and tenth), with interventions offered at different times over the course of nearly 6 months, in 12 schools and 65 classrooms, with a relatively short timeframe between pretest and posttest (14 to 27 days). Thus, it was implausible (though not impossible) that an external factor outside of the study was responsible for this finding.

We therefore considered effects that may have resulted from the study procedures. It was possible that participants’ responses at posttest were affected by completing the same items at pretest, either due to guessing and wanting to affect the study hypotheses or simple item-related biases introduced by familiarity with the questions [45]. However, some evidence suggests that questionnaire items do not affect student behavior [46]. Further, if this were the case, we would expect to have seen similar effects for cyberbullying victimization, bullying perpetration, and SEC, which we did not. We were also hesitant to ascribe these findings to regression to the mean [47]. The decrease was observed only for a specific subset of variables; as was already noted, bullying is common in school-based settings, so the likelihood that a large group of students from disparate settings and grades would significantly deviate from the population mean for bullying frequency is not conceptually strong. In addition, the impact of outlier cases on bullying frequency was minimized by the design, since the instance count in the questionnaire terminates at 4 (“4 or more times”). We also carefully avoided biases at the level of trial design by developing a protocol according to SPIRIT 2013 guidelines [48], preregistering the study, reporting even minor deviations, and attending to common sources of bias in clinical and prospective studies [49], though the possibility of unexplored confounding bias always must be considered [50]. Finally, 110 students in the control group indicated that they had seen a play or presentation by ACT Out! Ensemble before, though the degree of confounding influence, or the topic of prior performances, is unknown.

One such potential source of variance may have been the decision to randomize at the level of the classroom rather than at the level of the school. As has long been established, rigorous studies and evaluation of school-based programs is very methodologically difficult [51]. Prior research has indicated that randomizing at the school level can be problematic because schools often implement various SEL, SEC, and bullying programs, and so confounding variance can be introduced [23]. We were especially concerned about this when developing the protocol because SEL is written into expectations for Indiana schools, but not prescriptively (eg, schools can address it in different ways) [2]. By randomizing classrooms, we attempted to evade this problem by ensuring that a school’s other activities outside of this study were relatively equally represented among intervention and control clusters. However, it is important to consider that bullying does not occur within pre-existing clusters; that is, one is not limited to bullying or being bullied by students in the same homeroom period or English class. Thus, it is possible that an intervention affecting a bullying-related behavior, delivered to a random half of clusters within a grade and school, would have an effect on all clusters.

If this were the core mechanism explaining our data, then both bullying perpetration and victimization would theoretically be reduced; however, only victimization was reported to have been lowered. Thus, after careful consideration of the findings, including lack of SEC effect, high student receptivity and intervention fidelity, and significant time effects for traditional bullying victimization only, we hypothesize that the ACT Out! intervention may engender a heightened, defensive bystander response in participants. Bystander intervention in bullying has been associated with self-efficacy (belief that an intervention can be successful) [52], perceived knowledge about how one might successfully intervene [53], and the degree to which a bullying event is interpreted as serious [54]. Many of the scenarios for each grade level emphasized the roles of individuals other than the bully and the victim within the vignette and illustrated ways that others could intervene in a situation. They also identified potentially serious consequences that could emerge from bullying, including self-harm, so we infer surface-level plausibility of this explanation.

It is important to emphasize that while the data from this study were consistent with this explanation, the study itself *does not* provide causal evidence that the intervention engenders an increased likelihood for bystanders to intervene in bullying, nor was this an initial study hypothesis. Rather, we only know definitively that reported victimization decreased over time, in aggregate, among all participants. Additional research will be needed to determine the mechanism(s) by which this occurred. However, it is important to interpret and acknowledge all study findings to promote transparent research literature, and we have attempted to do so here.

### Limitations and Strengths

This study was truncated unexpectedly by the COVID-19 pandemic, which had the effect of preventing planned 3-month outcome data collection and moderately affecting the number of clusters available for analysis for short-term outcomes (loss of 11 clusters). There were also several unplanned deviations from the study protocol, each of which has been documented in Multimedia Appendix 1. Participating schools were from both urban and rural counties in Indiana, and student participants were generally more diverse than the population of Indiana as a whole. However, some caution should be used when generalizing these findings outside of the participating schools, especially since participating schools were those that volunteered to participate in a randomized trial. Further, given sample proportions, the results can be generalized more readily to middle and high school students than elementary school students. The study also had several notable strengths, including prespecification of all analyses; use of multiple objective, external consultants to the research team; thorough documentation of all protocol deviations; use of validated measures; and provision of all student documents and data in an open-source format.

### Conclusions and Next Steps

This study found no superiority for a 1-hour ACT Out! intervention compared to treatment as usual for SEC or offline bullying, but some evidence of a small effect for cyberbullying. As was already indicated, SEL and bullying interventions in schools tend to be lengthy and involved, and bullying interventions in particular may struggle to demonstrate effectiveness in randomized trials [24]. Since ACT Out! is much shorter and highly scalable, we interpreted the findings in this study, though few and small in magnitude, with interest.

We suggest several next steps for research in this area. First, a rigorous follow-up study with a new sample would be valuable, which addresses issues related to the interpretability of bullying victimization data, including measures of possible bystander effects and randomization at the school level while, if feasible, controlling for ongoing and recent bullying prevention programs. In doing so, scenario emphasis might also be reasonably shifted toward cyberbullying and away from physical bullying, for which potential iatrogenic effects in perpetration were computed in one of the models, though overall physical victimization declined. Second, additional data might also be collected on the sustainability of the effects beyond 2 weeks as well as on whether there is a dose-response relationship (eg, “Would 2 performances within a semester more strongly reduce victimization?”). This could also be extended by collecting measures related more broadly to student mental health, academic performance, and perceived school climate. Finally, on the practical side, given the high intervention fidelity, high student receptiveness, and preliminary evidence related to cyberbullying victimization, it would not be unreasonable for CMP to offer a performance of scenarios focused on cyberbullying prevention to supplement, rather than replace, extant bullying prevention programming. In practice (eg, outside of a trial), this intervention has comparatively low fiscal cost, only 1 hour of time is utilized, it requires no teacher time or preparation, and it may have some benefits.

### Additional Resources

To facilitate replication and transparent research processes, we have included supplemental files that may be valuable to researchers. These include a table of intracluster correlations (Multimedia Appendix 6), as well as the analysis syntax and datasets for per-protocol and intention-to-treat analyses (Multimedia Appendices 7-9, respectively).

## Appendix

Multimedia Appendix 1 Deviations from protocol.

Multimedia Appendix 2 ACT Out! scenario guide.

Multimedia Appendix 3 Fourth-grade fidelity instrument.

Multimedia Appendix 4 Seventh-grade fidelity instrument.

Multimedia Appendix 5 Tenth-grade fidelity instrument.

Multimedia Appendix 6 Intracluster correlation table.

Multimedia Appendix 7 Analysis syntax.

Multimedia Appendix 8 Dataset for per-protocol analysis.

Multimedia Appendix 9 Dataset for intent-to-treat analysis.

Multimedia Appendix 10 CONSORT E-HEALTH checklist (V 1.6.1).

## Abbreviations

BCS-A: Bullying and Cyberbullying Scale for Adolescents
CMP: Claude McNeal Productions
DSECS-S: Delaware Social-Emotional Competency Scale
ITT: intention to treat
MI: multiple imputation
PP: per-protocol
SEC: social-emotional competence
SEL: social-emotional learning
WCSD-SECA: Washoe County School District Social-Emotional Competency Assessment

## References

[1] ACT Out Ensemble 2020. McNeal C.

[2] Agley J, Jayawardene W, Jun M, Agley DL, Gassman R, Sussman S, Xiao Y, Dickinson SL (2020). Effects of the ACT OUT! Social Issue Theater Program on Social-Emotional Competence and Bullying in Youth and Adolescents: Protocol for a Cluster Randomized Controlled Trial. JMIR Res Protoc.

[3] Show licensing 2020. McNeal C.

[4] Tolan P, Ross K, Arkin N, Godine N, Clark E (2016). Toward an integrated approach to positive development: Implications for intervention. Applied Developmental Science.

[5] Bonell C, Hinds K, Dickson K, Thomas J, Fletcher A, Murphy S, Melendez-Torres GJ, Bonell C, Campbell R (2016). What is positive youth development and how might it reduce substance use and violence? A systematic review and synthesis of theoretical literature. BMC Public Health.

[6] Taylor RD, Oberle E, Durlak JA, Weissberg RP (2017). Promoting Positive Youth Development Through School-Based Social and Emotional Learning Interventions: A Meta-Analysis of Follow-Up Effects. Child Dev.

[7] Domitrovich CE, Durlak JA, Staley KC, Weissberg RP (2017). Social-Emotional Competence: An Essential Factor for Promoting Positive Adjustment and Reducing Risk in School Children. Child Dev.

[8] Espelage DL, Low S, Polanin JR, Brown EC (2013). The Impact of a Middle School Program to Reduce Aggression, Victimization, and Sexual Violence. Journal of Adolescent Health.

[9] Portnow S, Downer JT, Brown J (2018). Reductions in aggressive behavior within the context of a universal, social emotional learning program: Classroom- and student-level mechanisms. Journal of School Psychology.

[10] Modecki KL, Minchin J, Harbaugh AG, Guerra NG, Runions KC (2014). Bullying Prevalence Across Contexts: A Meta-analysis Measuring Cyber and Traditional Bullying. Journal of Adolescent Health.

[11] Arseneault L (2017). Annual Research Review: The persistent and pervasive impact of being bullied in childhood and adolescence: implications for policy and practice. J Child Psychol Psychiatr.

[12] Schoeler T, Duncan L, Cecil CM, Ploubidis GB, Pingault J (2018). Quasi-experimental evidence on short- and long-term consequences of bullying victimization: A meta-analysis. Psychological Bulletin.

[13] Gaffney H, Farrington DP, Ttofi MM (2019). Examining the Effectiveness of School-Bullying Intervention Programs Globally: a Meta-analysis. Int Journal of Bullying Prevention.

[14] Gaffney H, Ttofi MM, Farrington DP (2019). Evaluating the effectiveness of school-bullying prevention programs: An updated meta-analytical review. Aggression and Violent Behavior.

[15] Ttofi MM, Farrington DP (2010). Effectiveness of school-based programs to reduce bullying: a systematic and meta-analytic review. J Exp Criminol.

[16] Gaffney H, Farrington DP, Espelage DL, Ttofi MM (2019). Are cyberbullying intervention and prevention programs effective? A systematic and meta-analytical review. Aggression and Violent Behavior.

[17] Jiménez-Barbero JA, Ruiz-Hernández JA, Llor-Zaragoza L, Pérez-García M, Llor-Esteban B (2016). Effectiveness of anti-bullying school programs: A meta-analysis. Children and Youth Services Review.

[18] Menesini E, Salmivalli C (2017). Bullying in schools: the state of knowledge and effective interventions. Psychology, Health & Medicine.

[19] Bradshaw CP (2015). Translating research to practice in bullying prevention. American Psychologist.

[20] Serwacki M, Nickerson A, Schrantz M (2017). Guide to School-Wide Bullying Prevention Programs. Alberti Center for Bullying Abuse Prevention: University at Buffalo.

[21] Rawlings JR, Stoddard SA (2019). A Critical Review of Anti‐Bullying Programs in North American Elementary Schools. J School Health.

[22] Renshaw TL, Jimerson SR (2011). Enhancing Student Attitudes via a Brief, Universal-Level Bullying Prevention Curriculum. School Mental Health.

[23] Axford N, Bjornstad G, Clarkson S, Ukoumunne OC, Wrigley Z, Matthews J, Berry V, Hutchings J (2020). The Effectiveness of the KiVa Bullying Prevention Program in Wales, UK: Results from a Pragmatic Cluster Randomized Controlled Trial. Prev Sci.

[24] Rapee RM, Shaw T, Hunt C, Bussey K, Hudson JL, Mihalopoulos C, Roberts C, Fitzpatrick S, Radom N, Cordin T, Epstein M, Cross D (2020). Combining whole‐school and targeted programs for the reduction of bullying victimization: A randomized, effectiveness trial. Aggr Behav.

[25] Lauby JL, LaPollo AB, Herbst JH, Painter TM, Batson H, Pierre A, Milnamow M (2010). Preventing AIDS through Live Movement and Sound: Efficacy of a Theater-Based HIV Prevention Intervention Delivered to High-Risk Male Adolescents in Juvenile Justice Settings. AIDS Education and Prevention.

[26] Salmivalli Christina, Kaukiainen Ari, Voeten Marinus (2005). Anti-bullying intervention: implementation and outcome. Br J Educ Psychol.

[27] Joronen K, Konu A, Rankin HS, Astedt-Kurki P (2011). An evaluation of a drama program to enhance social relationships and anti-bullying at elementary school: a controlled study. Health Promotion International.

[28] Mantz LS, Bear GG, Yang C, Harris A (2016). The Delaware Social-Emotional Competency Scale (DSECS-S): Evidence of Validity and Reliability. Child Ind Res.

[29] Thomas HJ, Scott JG, Coates JM, Connor JP (2018). Development and validation of the Bullying and Cyberbullying Scale for Adolescents: A multi-dimensional measurement model. Br J Educ Psychol.

[30] Dent CW, Sussman S, Hennesy M, Galaif ER, Stacy AW, Moss M, Craig S (1998). Implementation and Process Evaluation of a School-Based Drug Abuse Prevention Program: Project towards No Drug Abuse. J Drug Educ.

[31] Davidson LA, Crowder MK, Gordon RA, Domitrovich CE, Brown RD, Hayes BI (2018). A continuous improvement approach to social and emotional competency measurement. Journal of Applied Developmental Psychology.

[32] Haahr M, Haahr S (2020). Random.org.

[33] Audette LM, Hammond MS, Rochester NK (2019). Methodological Issues With Coding Participants in Anonymous Psychological Longitudinal Studies. Educational and Psychological Measurement.

[34] Agley J, Tidd D, Jun M, Eldridge L, Xiao Y, Sussman S, Jayawardene W, Agley D, Gassman R, Dickinson SL (2020). Developing and Validating a Novel Anonymous Method for Matching Longitudinal School-Based Data. Educational and Psychological Measurement.

[35] White IR, Royston P, Wood AM (2010). Multiple imputation using chained equations: Issues and guidance for practice. Statist. Med.

[36] Wasserstein RL, Lazar NA (2016). The ASA Statement on -Values: Context, Process, and Purpose. The American Statistician.

[37] Jakobsen JC, Gluud C, Wetterslev J, Winkel P (2017). When and how should multiple imputation be used for handling missing data in randomised clinical trials – a practical guide with flowcharts. BMC Med Res Methodol.

[38] Ranganathan P, Pramesh C, Aggarwal R (2016). Common pitfalls in statistical analysis: Intention-to-treat versus per-protocol analysis. Perspect Clin Res.

[39] Bickman L, Riemer M, Brown JL, Jones SM, Flay BR, Li KK, DuBois D, Pelham Jr W, Massetti GM (2009). Approaches to measuring implementation fidelity in school-based program evaluations. Journal of Character Education.

[40] Rohrbach LA, Gunning M, Sun P, Sussman S (2009). The Project Towards No Drug Abuse (TND) Dissemination Trial: Implementation Fidelity and Immediate Outcomes. Prev Sci.

[41] Little MA, Sussman S, Sun P, Rohrbach LA (2013). The effects of implementation fidelity in the Towards No Drug Abuse dissemination trial. Health Education.

[42] McKown C (2019). Student social and emtoional competence assessment: The current state of the field and a vision for its future. Collaborative for Academic, Social, and Emotional Learning.

[43] Salkind NJ (2010). Encyclopedia of Research Design.

[44] Tokunaga RS (2010). Following you home from school: A critical review and synthesis of research on cyberbullying victimization. Computers in Human Behavior.

[45] Choi BCK, Pak AWP (2005). A catalog of biases in questionnaires. Prev Chronic Dis.

[46] Sussman S, Dent C, Burton D, Stacy A, Flay B (1995). Developing school-based tobacco use prevention and cessation programs.

[47] Bland JM, Altman DG (1994). Statistics Notes: Some examples of regression towards the mean. BMJ.

[48] Chan A, Tetzlaff J, Gøtzsche Peter C, Altman D, Mann H, Berlin J, Dickersin K, Hróbjartsson A, Schulz KF, Parulekar WR, Krleza-Jeric K, Laupacis A, Moher D (2013). SPIRIT 2013 explanation and elaboration: guidance for protocols of clinical trials. BMJ.

[49] Gluud LL (2006). Bias in clinical intervention research. Am J Epidemiol.

[50] Stenson JF, Kepler CK (2019). Bias in Prospective Research and How to Avoid it. Clinical Spine Surgery.

[51] Jaycox LH, McCaffrey DF, Ocampo BW, Shelley GA, Blake SM, Peterson DJ, Richmond LS, Kub JE (2016). Challenges in the Evaluation and Implementation of School-Based Prevention and Intervention Programs on Sensitive Topics. American Journal of Evaluation.

[52] Thornberg R, Jungert T (2013). Bystander behavior in bullying situations: Basic moral sensitivity, moral disengagement and defender self-efficacy. Journal of Adolescence.

[53] Van Cleemput K, Vandebosch H, Pabian S (2014). Personal characteristics and contextual factors that determine "helping," "joining in," and "doing nothing" when witnessing cyberbullying. Aggress Behav.

[54] Jenkins LN, Nickerson AB (2016). Bullying participant roles and gender as predictors of bystander intervention. Aggr. Behav.

