# Supplementary material for: Effects of ACT Out! Social Issue Theater on Social-Emotional Competence and Bullying in Youth and Adolescents: Cluster Randomized Controlled Trial
Source: JMIR Ment Health. 2021 Jan 6;8(1):e25860. doi: 10.2196/25860 (PMC7817353; doi:10.2196/25860)
Supplement: Multimedia Appendix 1 [file mental_v8i1e25860_app1.docx]

*Deviations from Protocol - Design*

One school was only able to participate if students were randomly assigned to clusters by the research team (e.g., preexisting classrooms that could participate in the intervention or take the survey [control] did not exist because of the way the school’s schedule was designed). Thus, for this school, we randomly assigned individual students to eight equivalent clusters and then randomly assigned four of those clusters to the intervention, and four to the control arm. Students received the intervention in clusters per protocol.

*Deviations from Protocol – Participants*

The study protocol specified that all schools would be recruited from Marion County, IN. However, many schools displayed reluctance to participate in a cRCT, so we expanded recruitment to the state of Indiana using convenience and snowball methods until our planned sample threshold was achieved. Because this extended our recruitment timeline, we formally changed the registered protocol on November 14, 2019 to reflect a three-month rather than six-month follow-up period (though, as described later, COVID-19 obviated this decision).

*Deviations from Protocol – Outcomes*

The following planned outcomes and sub-outcomes were not collected as a result of the COVID-19 pandemic: referrals to discipline for bullying, truancy, absenteeism, and academic performance (all of which were to have been measured at three-month posttest only), in addition to the three-month posttest data for the other primary and secondary outcomes.

*Deviations from Protocol – Quality Control*

On January 27^th^, the primary individual conducting fidelity checks left the project. As a result, members of the research team conducted the remainder of the fidelity checks for interventions. In addition, the protocol indicated that the Kappa statistic would be used to calculate interrater reliability. However, the level of agreement (and overall fidelity) was extremely high, resulting in a level of trait prevalence (e.g., conforming to the checklist) that substantively suppressed the ability of Kappa to accurately indicate interrater reliability (Gwet 2002). Thus, only the percentile agreement between raters was computed. Finally, although fidelity documents were created for all interventions that were delivered, all documentation was done on paper, and six of the forms were unable to be located prior to data entry. Thus, we report extant fidelity data for 34 interventions (though anecdotally note that data from the missing forms were consistent with those we report).

*Deviations from Protocol – Matching*

The protocol indicated that we would use computerized matching and then manual inspection, but also implied that the computerized system would use one-off matching. The system we developed is similar but uses weighted probability ranked matching instead of one-off matching. As indicated, we produced a full paper detailing the process (Agley et al. 2020b).

*Deviations from Protocol – Analysis*

The protocol indicated that unmatched posttest surveys would not be included in the analysis. However, on review by multiple statistical consultants, it was determined that unmatched pretests and posttests would be assigned unique match values, treated as unpaired, and analyzed within their originally assigned arm.
