# Supplementary material for: Effects of ACT Out! Social Issue Theater on Social-Emotional Competence and Bullying in Youth and Adolescents: Cluster Randomized Controlled Trial
Source: JMIR Ment Health. 2021 Jan 6;8(1):e25860. doi: 10.2196/25860 (PMC7817353; doi:10.2196/25860)
Supplement: Multimedia Appendix 2 [file mental_v8i1e25860_app2.docx]

**ACT OUT! Scenario and Audience Interplay Guide**

*Note: Because ACT OUT! is an improvisational theatrical performance, these guidelines serve to summarize the raw content of each scenario and questions that are posed by the facilitator to influence audience conversation. However, these descriptions do not attempt to capture the underlying execution of the scenarios, which is undertaken by professionally-trained dramatists.*

_____________________________________________________________________________________

**4^th^ Grade**

**Body Language** (social awareness)

The facilitator starts out by asking if the students think they can tell what someone is thinking or feeling just by looking at them. Then, one by one, the actors stand in front and come up with a pose. Students are asked to guess what the actors are thinking or feeling based on the poses.

After the facilitator gets a few answers, she/he asks what students might potentially say to someone who looked like that (e.g., what power do we have to influence others?).

*Example Discussion Questions*:

How many of you take the time to stop and think about how someone else is feeling?

Does this person look like they might need help? Why?

**Chair** (relationship skills, self-management)

Ann starts to sit in chair and realizes she forgot her backpack. Zach rushes past her and steals her chair. Ann starts to yell at him and tries to push Zach out of the chair; he yells back and calls her names.

*Example Discussion Questions:*

Who is in control?

How do you think she should have approached this conflict?

What do you think it takes to solve a conflict?

**Rumor** (responsible decision-making, self-management)

Zach tells Ann that DeNay has been saying mean things about her and that she is going to get in trouble (really hyping Ann up). DeNay walks across the stage, and Zach encourages Ann to do something about it. Ann pushes DeNay, DeNay pushes back.

*Example Discussion Questions:*

Who has the power in this scenario?

Who is blaming someone else for his or her actions?

Who is not taking responsibility at all?

**Girls’ Exclusion** (social awareness, relationship skills)

Tess opens the scene by showing that she is very upset and walking away from Ann. Ann tells her to stop walking away because “the other girls were just joking.” Tess explains that the other girls make fun of her every day and today, they talked about going to the mall and they purposely did not invite her. Ann tells her that she (Tess) needs to pretend to like the things that they like in order to fit in and that it is her fault they treat her like that.

*Example Discussion Questions:*

Do you have to be friends with everyone?

Who has the power to do something?

What is the difference between an up stander and a bystander?

**Weapon** (responsible decision-making, relationship skills)

Zach comes over to Ann’s house after school very upset. He tells her that Jake and Sam have been messing with him again and that he has to do something about it. She agrees, but doesn’t know what he can do. Zach asks to borrow Ann’s mom’s gun. He says he won’t even load it, he just wants to scare them. He promises he will bring the gun back and she won’t get into trouble.

*Example Discussion Questions:*

What is the worst thing that could happen?

Do you think that Zach stopped to think this all the way through?

Should Ann say “Yes” to Zach’s plan? Why or why not?

Do you think they should go to an adult?

**7^th^ Grade**

**Chair** (relationship skills, self-management)

Ann starts to sit in chair and realizes she forgot her backpack. Zach rushes past her and steals her chair. Ann starts to yell at him and tries to push Zach out of the chair; he yells back and calls her names.

*Example Discussion Questions:*

Who is in control?

How do you think she should have approached this conflict?

What do you think it takes to solve a conflict?

**Disruptive Student** (social awareness, self-management, relationship skills)

Tessa (the teacher) starts class. Zach enters class late, talks loudly with other students, touches their belongings, and is generally disruptive. Tessa tells him that he has to do his report first today. Zach gets mad. Ann, another student, has been overreacting and laughing at everything that Zach does. Tessa finally gets frustrated and tells him to go to the office. Zach becomes very belligerent and kicks a chair on the way out.

*Example Discussion Questions:*

Did Zach affect anyone else?

What is Zach responsible for and why?

Why should you care about how others feel/view you?

**Cyberbullying** (*all categories*)

Ann and DeNay are walking down the hall, and Ann is making fun of people. When she sees Zach, she shows him a picture that she made of him in a dress with a caption that says “Gay all day.” She also points out the number of likes that she is getting. Zach is upset and begs her to take it down. DeNay halfheartedly laughs along but does try to get Ann to stop. Then Ann turns on her (DeNay), so DeNay joins in on making fun of Zach.

*Example Discussion Questions:*

Is it ok to post whatever you want on the Internet?

Based on Zach’s expression, how do you think he feels?

Who could have done something to affect this situation?

**Cutting** (relationship skills)

Tess visits Ann’s house and finds her (Ann) up in her room in the dark. Tess asks her why she didn’t show up to cheer try outs. Ann tells her she’s sick of Jen and Heidi making fun of her. Tess tries to tell her that they don’t mean anything by it, then Ann shows her a text that Jen sent telling her to kill herself because Ann is a freak. Tess asks her to tell her mom; Ann gets upset and wants Tess to leave. Tess grabs Ann’s arm and notices that Ann has been cutting herself.

*Example Discussion Questions:*

What are healthy ways of dealing with stress?

Do you think Ann can handle this on her own or talk to someone/ who?

What other way could she handle this problem?

**Park Beat Down** (responsible decision-making)

Zach is just about to get on the bus to go home after school. Ann stops him and tells him that Ryan and Jason are jumping Jim in the park. Zach just got in trouble because of Jim and would love to see it but does not want to get in trouble. Ann tells him they are just going to video it and leave right away so they won’t get in trouble.

*Example Discussion Questions:*

What else could happen if they decide to go?

Is Ann being a good friend?

Would they get in trouble at school?

**10^th^ Grade**

**Candy** (self-management, social awareness)

Ann is walking down the hall when Josiah asks her for a piece of candy. She says she doesn’t have any and proceeds to touch her invasively while looking for it. Ann laughs but tries to move away; at the same time, she tells him to stop, but not forcefully.

*Example Discussion Questions:*

Does Ann look like she likes what Josiah did?

Do you think Josiah meant to make Ann uncomfortable?

How could Ann communicate to Josiah that she was uncomfortable?

**Sexting** (responsible decision making, social awareness)

Ann walks up to Tess in the hallway and tells her she’s mad at her. Tess asks Ann what is going on, because everyone has been treating her differently today. Ann tells Tess that Zach sent the naked picture that Tess sent to him to other people. Ann calls her a slut and tells her that she doesn’t want to be friends with her anymore. Zach enters the scene and Tess asks him to delete the photo. Zach says he will if she gives him a private show.

*Example Discussion Questions:*

Do you think Zach stopped to think how this would affect Tess?

Did Tess think through what might happen if she sent that picture?

What do you think Ann is thinking when she criticizes Tess?

Do you think she needs help to deal with this, and how does she get her power back?

**Cyberbullying** (*all categories*)

Ann and DeNay are walking down the hall, and Ann is making fun of people. When she sees Zach, she shows him a picture that she made of him in a dress with a caption that says “Gay all day.” She also points out the number of likes that she is getting. Zach is upset and begs her to take it down. DeNay halfheartedly laughs along but does try to get Ann to stop. Then Ann turns on her (DeNay), so DeNay joins in on making fun of Zach. After Ann leaves, DeNay tells Zach should just come out. Zach says that he’s not gay and that he will take of it. Zach’s last comment is that “no one will have to worry about me tomorrow.”

*Example Discussion Questions:*

What did Zach mean when he said that last line? What should DeNay do about it?

Why do you think DeNay is not standing up to Ann?

Why do bullies act the way they do?

**Stolen Hoodie** (self-management, responsible decision making)

Zach and Josiah skipped the last class period of the day and went to the mall. Zach put a hoodie he didn’t pay for into Josiah’s backpack; they are now in the mall security office waiting for their parents to show up. Josiah is mad at Zach for what he did, but Zach tells him to relax because it is not a big deal. Josiah’s mom shows up Zach leaves. Josiah’s mom goes off on him and tells him he is “worthless, just like his father.” Josiah refuses to turn Zach in.

*Example Discussion Questions:*

How could Josiah have handled himself differently?

What could have prevented this from happening?

Do you think Josiah and his mom have a healthy relationship?

**Cup Game** (social awareness)

The facilitator calls three student volunteers to the front of the room. Two students stand at either end of the room (each with two cups), and the third student stands in the middle. The student in the middle has one cup and is told that he or she can’t physically touch other cups, except with the original cup. The goal of the game is for the student to walk back and forth, creating and balancing a stack of cups from alternating sides. However, the actors try to distract the student in order to make the cups fall. The facilitator discusses this game as a metaphor for goals and dreams.

*Example Discussion Questions:*

What does it take to overcome obstacles?

Did the students on either side help the student in the middle, or were they passive bystanders?

Did the student in the middle ask for help?

How do we respond to frustration?
