# Supplementary material for: Effects of ACT Out! Social Issue Theater on Social-Emotional Competence and Bullying in Youth and Adolescents: Cluster Randomized Controlled Trial
Source: JMIR Ment Health. 2021 Jan 6;8(1):e25860. doi: 10.2196/25860 (PMC7817353; doi:10.2196/25860)
Supplement: Multimedia Appendix 3 [file mental_v8i1e25860_app3.docx]

***ACT OUT! Fidelity Checklist (4^th^)***

Classroom ID:

Raters should clearly indicate whether each study element is either **present** (occurred during the specific performance) or **absent** (did not occur during the specific performance).

| Body Language | Present | Absent |
| --- | --- | --- |
| Facilitator asks students to guess what actors are thinking / feeling based on their poses. |  |  |
| Facilitator asks at least one general question about thinking about how others might be feeling. |  |  |
| Facilitator asks at least one specific question about whether an actor looks like they may need help. |  |  |

| Chair | Present | Absent |
| --- | --- | --- |
| Scenario includes an example of name-calling. |  |  |
| Scenario includes an example of pushing. |  |  |
| Facilitator asks at least one specific question about how Ann should have approached the conflict. |  |  |
| Facilitator asks at least one general question about how conflicts can be solved. |  |  |

| Rumor | Present | Absent |
| --- | --- | --- |
| Scenario includes an example of gossip. |  |  |
| Scenario includes an example of pushing. |  |  |
| Facilitator asks at least one specific question about who holds the power in this scenario. |  |  |
| Facilitator asks at least one specific question about blaming and responsibility related to the scenario. |  |  |

| Girls’ Exclusion | Present | Absent |
| --- | --- | --- |
| Scenario includes an example of social exclusion. |  |  |
| Facilitator asks at least one specific question about who could do something to improve this situation. |  |  |
| Facilitator asks at least one general question about friendship. |  |  |
| Facilitator asks at least one general question about standing up for people versus being a bystander. |  |  |

| Weapon | Present | Absent |
| --- | --- | --- |
| Scenario includes an example of students attempting to access a parent’s gun. |  |  |
| Facilitator asks at least one specific question about what could potentially happen. |  |  |
| Facilitator asks at least one specific question about whether Zach thought through his plan. |  |  |
| Facilitator asks at least one specific question about whether Zach and Ann should talk to an adult about their plan. |  |  |
