# Supplementary material for: Effects of ACT Out! Social Issue Theater on Social-Emotional Competence and Bullying in Youth and Adolescents: Cluster Randomized Controlled Trial
Source: JMIR Ment Health. 2021 Jan 6;8(1):e25860. doi: 10.2196/25860 (PMC7817353; doi:10.2196/25860)
Supplement: Multimedia Appendix 4 [file mental_v8i1e25860_app4.docx]

***ACT OUT! Fidelity Checklist (7^th^)***

Classroom ID:

Raters should clearly indicate whether each study element is either **present** (occurred during the specific performance) or **absent** (did not occur during the specific performance).

| Chair | Present | Absent |
| --- | --- | --- |
| Scenario includes an example of name-calling. |  |  |
| Scenario includes an example of pushing. |  |  |
| Facilitator asks at least one specific question about how Ann should have approached the conflict. |  |  |
| Facilitator asks at least one general question about how conflicts can be solved. |  |  |

| Disruptive Student | Present | Absent |
| --- | --- | --- |
| Scenario includes an example of disruptive student behavior. |  |  |
| Scenario includes an example of one student affecting another student’s behavior. |  |  |
| Facilitator asks at least one specific question about whether Zach affected anyone else. |  |  |
| Facilitator asks at least one general question about why a person should care how others feel about them or view them. |  |  |

| Cyberbullying | Present | Absent |
| --- | --- | --- |
| Scenario includes an example of cyberbullying. |  |  |
| Scenario includes an example of peer pressure influencing someone to tease someone else. |  |  |
| Facilitator asks at least one general question about what is ok to post on the Internet. |  |  |
| Facilitator asks at least one specific question asking students to interpret how Zach feels based on his body language or expression. |  |  |
| Facilitator asks at least one specific question related to who could intervene in this scenario. |  |  |

| Cutting | Present | Absent |
| --- | --- | --- |
| Scenario includes an example of bullying using text messages. |  |  |
| Scenario includes an example of self-harm. |  |  |
| Facilitator asks at least one general question about healthy ways to deal with stress. |  |  |
| Facilitator asks at least one specific question about how Ann could potentially address this situation. |  |  |

| Park Beat Down | Present | Absent |
| --- | --- | --- |
| Scenario contains a discussion of whether or not to go watch a fight after school. |  |  |
| Facilitator asks a specific question about potential consequences for Zach and Ann if they go. |  |  |
| Facilitator asks at least one specific question about whether Zach and Ann could get in trouble for videotaping the fight. |  |  |
