# Supplementary material for: Effects of ACT Out! Social Issue Theater on Social-Emotional Competence and Bullying in Youth and Adolescents: Cluster Randomized Controlled Trial
Source: JMIR Ment Health. 2021 Jan 6;8(1):e25860. doi: 10.2196/25860 (PMC7817353; doi:10.2196/25860)
Supplement: Multimedia Appendix 5 [file mental_v8i1e25860_app5.docx]

***ACT OUT! Fidelity Checklist (10^th^)***

Classroom ID:

Raters should clearly indicate whether each study element is either **present** (occurred during the specific performance) or **absent** (did not occur during the specific performance).

| Candy | Present | Absent |
| --- | --- | --- |
| Scenario includes an example of a male student making a female student physically uncomfortable. |  |  |
| Facilitator asks at least one specific question about whether Ann liked what Josiah did. |  |  |
| Facilitator asks at least one specific question about whether Josiah meant to make Ann uncomfortable. |  |  |

| Sexting | Present | Absent |
| --- | --- | --- |
| Scenario includes an example of a private picture being shared without the subject’s consent. |  |  |
| Scenario includes an example of a female being shamed or insulted because someone else shared her private picture. |  |  |
| Facilitator asks at least one specific question about whether Zach thought about how sharing the picture would affect Tess. |  |  |
| Facilitator asks at least one specific question about Tess’s thoughts and motivations for sending the picture. |  |  |
| Facilitator asks at least one specific question about Ann’s motivations and behavior. |  |  |

| Cyberbullying | Present | Absent |
| --- | --- | --- |
| Scenario includes an example of cyberbullying. |  |  |
| Scenario includes an example of peer pressure influencing someone to tease someone else. |  |  |
| Scenario includes an example of a bullied student implying that he might harm himself in response to the bullying. |  |  |
| Facilitator asks at least one specific question about what to do when a friend suggests he/she might hurt him/herself. |  |  |
| Facilitator asks at least one specific question about why a bystander might not stand up for someone being bullied. |  |  |
| Facilitator asks at least one general question about why bullies act the way they do. |  |  |

| Stolen Hoodie | Present | Absent |
| --- | --- | --- |
| Scenario includes an example of one student creating legal trouble for another student. |  |  |
| Facilitator asks at least one specific question about what Josiah could do differently. |  |  |
| Facilitator asks at least one general question about what could have prevented this from happening. |  |  |
| Facilitator asks at least one specific question about whether Josiah and his mom have a healthy relationship. |  |  |

| Cup Game | Present | Absent |
| --- | --- | --- |
| Facilitator asks at least one general question about overcoming obstacles. |  |  |
| Facilitator asks at least one specific question about whether the students playing the game offered or solicited help. |  |  |
| Facilitator asks at least one general question about how we respond to frustration. |  |  |
