# Supplementary material for: Effects of ACT Out! Social Issue Theater on Social-Emotional Competence and Bullying in Youth and Adolescents: Cluster Randomized Controlled Trial
Source: JMIR Ment Health. 2021 Jan 6;8(1):e25860. doi: 10.2196/25860 (PMC7817353; doi:10.2196/25860)
Supplement: Multimedia Appendix 6 [file mental_v8i1e25860_app6.docx]

| **Intra-Cluster Correlations (ICC)** | | | | |
| --- | --- | --- | --- | --- |
| **Variable** | **ITT without MI** | **ITT with MI** | **PP without MI** | **PP with MI** |
| ***Bullying*** | - | - | - | - |
| *Victimization* | - | - | - | - |
| Physical | 0.0661 | 0.0675 | 0.0647 | 0.0679 |
| Verbal | 0.0555 | 0.0562 | 0.0517 | 0.0529 |
| Relational | 0.0499 | 0.0526 | 0.0451 | 0.0489 |
| Cyber | 0.0641 | 0.0652 | 0.0616 | 0.0637 |
| *Perpetration* | - | - | - | - |
| Physical | 0.0453 | 0.0448 | 0.0459 | 0.0477 |
| Verbal | 0.0411 | 0.0409 | 0.0421 | 0.0452 |
| Relational | 0.0410 | 0.0412 | 0.0412 | 0.0442 |
| Cyber | 0.0335 | 0.0366 | 0.0363 | 0.0381 |
| ***SEC*** | 0.0419 | 0.0433 | 0.0380 | 0.0423 |
| Social Awareness | 0.0311 | 0.0306 | 0.0356 | 0.0361 |
| Emotion Regulation | 0.0091 | 0.0098 | 0.0105 | 0.0120 |
| Relationship Skills | 0.0392 | 0.0377 | 0.0403 | 0.0385 |
| Responsible Decision-   Making | 0.0419 | 0.0409 | 0.0460 | 0.0447 |
